# Study on Chip Formation Mechanism of Single Crystal Copper Using Molecular Dynamics Simulations

**DOI:** 10.1186/s11671-022-03731-2

**Published:** 2022-09-19

**Authors:** Peng Zhang, Xinjian Li, Jiansheng Zhang, Yi Zhang, Xiaoguang Huang, Guigen Ye

**Affiliations:** College of Pipeline and Civil Engineering, China University of Petroleum (East China), Qingdao, China

**Keywords:** Single crystal copper, Nano-cutting, Chip formation mechanism, Molecular dynamics simulation

## Abstract

Nano-cutting is an important development direction of the modern manufacturing technology. However, the research on the mechanism underlying nano-cutting lags far behind the practical application, which restricts the development of this advanced manufacturing technology. The chip formation process is the basic process of nano-cutting, and it is of key importance for the mechanism research of nano-cutting. In this paper, the nano-tensile behavior of single crystal copper was studied based on the molecular dynamics simulations. The toughness and brittleness characteristics of the copper at different temperatures were analyzed. Then, the molecular dynamics simulations of nano-cutting for single crystal copper with different toughness and brittleness were studied. The crystal structure, cutting force, stress–strain distribution and atomic motion characteristics were systematically investigated. The nano-chip formation mechanism of single crystal copper was revealed. The results show that the chip is formed through two ways, namely the shear and extrusion. The material near the free surface of the workpiece undergoes continuous shear slip and periodic long-distance slippage along the primary shear direction, forming the block chip in which the FCC and HCP structures are orderly distributed. The material near the tool-chip interface is extruded by the tool, block chip and stagnation zone to form the flowing chip with amorphous structure. As the temperature increases, the occurrence frequency of long-distance slippage in the block chip increases, while the slippage degree decreases. Besides, with the increase in temperature, the thickness of block chip formed by shear slip decreases, while the thickness of flowing chip formed by extrusion increases.

## Introduction

Cutting is one of the basic methods for material removal. As early as the 1950s, some scholars studied the cutting mechanism. Some classical macro-cutting models, such as the simple shear model and card model, were proposed [1, 2]. With the continuous reduction of cutting size, cutting models and cutting methods are constantly improved [3–6]. Increasingly stringent machining requirements have reduced machining accuracy from the micron level in the early 1970s to the nanometer level today [7], which is nano-cutting. Nano-cutting has broad application prospects in optical instrument surface processing and nano-electromechanical system manufacturing. Besides, it is one of the most effective and economical methods to realize nano-surface processing [8]. However, the mechanical, thermodynamic, electrical and other basic properties of nanomaterials will change greatly under the influence of the size effect and interface effect when the scale of materials is reduced to the nanoscale. So, the original cutting mechanism at the macroscale is no longer applicable at the nanoscale [9, 10]. At present, research on nano-cutting mechanisms is still in its infancy, and the core of research lies in the chip formation mechanism. However, the chip formation mechanism is still unclear due to the different mechanical properties and cutting parameters of different materials. Therefore, the nano-cutting mechanisms need to be further studied.

The research methods of chip formation mechanism at the nanoscale are divided into experimental and molecular dynamics (MD) [11]. Compared with the expensive and complex experimental method, the MD method has the advantages of low cost, convenient calculation and observation of the cutting process in picoseconds (ps). In addition, with the development of computer science, the accuracy and scale of MD simulation are constantly improving, which has become an essential means to study micro- and nano-cutting mechanisms. Based on MD simulation, a series of researches on nano-cutting have been carried out by domestic and foreign scholars. In earlier studies, limited by the power of computers, the cutting model size and cutting depth are small [12]. Only a few atoms on the workpiece surface form chips. In the cutting simulation, only a few atoms on the workpiece surface form chips, which makes most of the chips lose their crystal structure under the action of tool-chip friction, and many details of the chip formation process cannot be presented. Since then, the computing power of computers has gradually improved, and the model size is no longer the main factor limiting the research on chip formation mechanism. Pei et al. [13] studied the influence of potential function on the cutting process of single crystal copper. It is found that the similar chip formation process of single crystal copper can be obtained by using either EAM or Morse potential. Since then, researchers have generally focused on the influence of cutting parameters on chip formation mechanism. Lin et al. [14, 15] also took single crystal copper as the research object and studied the influence of cutting speed on nano-cutting process at 293 K. It was found that the increase in cutting speed leads to the closer distribution of atoms in the chip. Wang et al. [16] divided the single crystal copper workpiece into many strip regions perpendicular to the cutting direction. The shearing chip formation process of atoms in front of the tool was observed. Xie et al. [17, 18] simulated the cutting process of single crystal copper at different cutting depths. It was found that the radius effect and size effect influenced the material removal rate greatly. In addition to studying the macroscopic structure change in nano-cutting process, scholars gradually paid attention to the crystal phase transition and crystal structure distribution, so as to summarize the chip formation mechanism. Sharma [19] simulated the cutting process of single crystal copper under different crystal orientations. The crystal structure distribution was analyzed. It was found that chips are more easily formed by shear in the (0 1 0) [${\overline{\text{1}}}$ 0 0] cutting direction. The dislocation distribution in chip was perpendicular to the cutting direction when the cutting direction is (${\overline{\text{1}}}$ 1 0) [1 1 0]. Wang et al. [20] studied the influence of fluid media on chip formation process of single crystal copper. It is found that the presence of water causes the hot zone in the chip to move from the tool-chip contact zone to the top of the chip. Zhang et al. [21] studied the influence of cutting speeds in the cutting process of single crystal copper. The distribution of dislocation and von Mises stress were analyzed. It was found that the dislocation cannot be fully extended and the number of dislocations in the chip is small under the high cutting speed. In addition to changes in crystal structure during chip formation, some researchers have also studied different atomic motion patterns and stress distribution at chip roots. Xu et al. [22] simulated the nano-cutting process of aluminum at 293 K. A triangular stagnation region was found in front of the rake face of the tool. The stagnation region divides the workpiece into chip and machined surface. The atoms in the stagnation region are basically at rest compared to the surrounding atoms. Wang [23] conducted molecular dynamics cutting simulation of single crystal copper. Through stress analysis, it was found that the workpiece atoms in front of the tool which were about to form chips were subjected to greater shear stress, while the atoms below the tool were subjected to compressive stress.

Among many influencing factors, cutting temperature can significantly affect the mechanical properties of materials and therefore has great influences on the cutting process. So the preheating and precooling technologies are usually used to improve the cutting performance. The liquid nitrogen cooling and laser-assisted technology are the most commonly used methods to reduce and increase cutting temperature, respectively. Liquid nitrogen cooling is often used in alloy processing. Lei et al. [24] simulated the cutting process of γ-TiAl alloy at low temperature. They found that the machining surface quality is better at low temperature than that at normal temperature. However, the effect of low temperature on the machining surface quality is limited, and the optimal cutting temperature is 173 K. Kayank et al. [25] and Jamil et al. [26] experimentally investigated the cutting process of alloys under low temperature and high pressure. They found that cutting force and tool wear reduce at lower temperature. Li et al. [27] used MD method to simulate the cutting process of TiAl in water medium environment. They stated that the cooling effect of water medium took away part of the heat in the cutting area, which can reduce the subsurface damage. The laser-assisted technology may also be beneficial for the metal cutting [28, 29]. Liu et al. [30] performed tension and cutting simulations of single crystal silicon at different temperatures and found that silicon changed from brittle to tough at 1700 K. The results showed that the stagnation zone and shear angle in front of the tool are small at high temperature, which promotes the plastic deformation of the material. Besides, the densification degree near the machined surface is greatly reduced at higher temperature, and better surface quality can be obtained. Luo et al. [31] simulated the cutting process of single crystal germanium at different temperatures. They showed that the increase in cutting temperature facilitates the generation of the internal dislocations in the workpiece, resulting in a decrease in the average cutting force. In order to further study the influence of temperature on the anisotropic cutting behavior of single crystal silicon, Saeed et al. [32–34] performed MD simulations of cutting single crystal silicon at different temperatures and crystal orientations. The anisotropy in the cutting forces, specific cutting energies, yielding stresses and temperatures were observed to be increased with the increase in cutting temperature.

It should be pointed out that the research on the influences of cutting temperature mainly focuses on the cutting force, machining surface and tool damage. The effect of cutting temperature on the chip formation process has been rarely studied. The underlying mechanism of the chip formation in nano-cutting at different temperatures is still unclear. This work will systematically study the nano-cutting behaviors of single crystal copper at different temperatures with different toughness and brittleness. The crystal structure, cutting force, stress–strain distribution and atomic motion characteristics will be analyzed in detail for different temperatures to reveal the underlying mechanism of chip formation of single crystal copper with different toughness and brittleness.

## Mechanical Properties at Different Temperatures

**Fig. 1:**
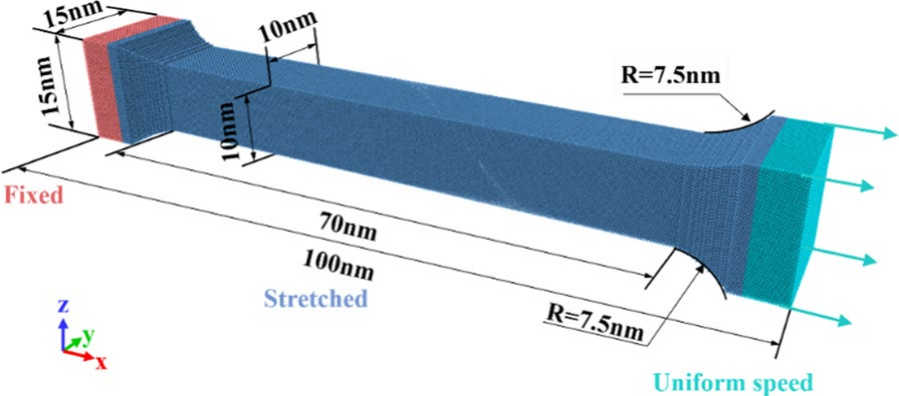
Nano-stretching model of single crystal copper

In order to obtain the single crystal copper samples with different toughness and brittleness. The samples is preheated and precooled at different temperatures. Meanwhile, in order to establish the relationship between the mechanical properties and temperature. The tensile model of single crystal copper (see Fig. 1) is established based on Lammps [35], referring to the macroscopic uniaxial tensile experiment. Then, the tensile behavior of single crystal copper at different temperatures is used to characterize its toughness and brittleness.

In the nano-tensile model, the side length of the square section in the middle of the model (stretched part) is 10 nm. The side length of the square section at both ends of the model is set as 15 nm to reduce the influence of the end effect on the simulation results. The connection between the two ends and the middle part of the specimen is smoothly transitioned by an arc with a radius of 7.5 nm, which can effectively reduce the stress concentration phenomenon. The interatomic interaction potential of Copper (Cu) is described by EAM potential [36]. Relaxation is required to improve the accuracy of simulation results, after the model is established. First, the conjugate gradient method is used to minimize the energy of the model. Then the Nosé-Hoover hot bath method is used to reach the initial temperature of the model under the NVT ensemble [37]. After the model reached a stable state through relaxation, the left end of the model is fixed and the right end is stretched along the axial direction with a constant speed. For the nano-tensile simulations, the relative velocity of the two ends of the model is usually set between 1 and 15 m/s [38–42]. In this work, an intermediate value of 10 m/s is selected for uniform stretching. During the stretching process, the boundary conditions in *x*, *y* and *z* directions are set as free boundaries. Verlet algorithm is used to numerically integrate Newton’s equation of motion [43]. In order to obtain a general conclusion, the studied temperature range is selected as far as possible to cover the interval from near absolute zero to near the melting point of single crystal copper (4 K–1200 K), and the selected temperature points (4 K, 300 K, 600 K, 900 K and 1200 K) are uniformly distributed in this temperature range. The detailed parameters of the model are shown in Table 1.

**Table 1:** Tensile simulation parameters

Tensile parameters	Value
Interatomic interaction	EAM
Model size (*x*, *y*, *z*)	70 nm, 15 nm, 15 nm
Number of workpiece atoms	1,072,564
Tensile speed	10 m/s
Tensile temperature	4 K, 300 K, 600 K, 900 K, 1200 K
Timestep	1 fs

**Fig. 2:**
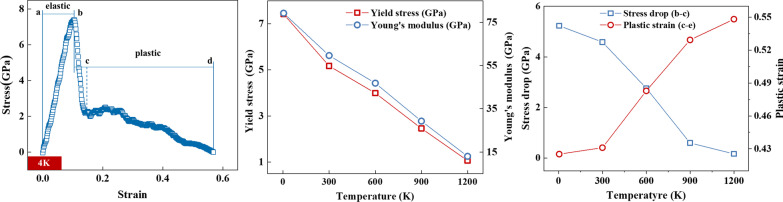
Tensile data **a** stress–strain curve at 4 K **b** Young’s modulus and yield stress, **c** stress drop and plastic strain

Firstly, the stress–strain curves of single crystal copper at different temperatures are extracted, as shown in Fig. 2a. The curve can be divided into four stages, namely a–b, b–c and c–d stages. The a–b is the elastic stage. Stress and strain show an excellent linear relationship in this stage. The specific surface area of metal crystals at the nanometer scale is much larger than that at the macroscopic scale. So, the electrons are concentrated on the surface of the crystal, and the metal bonds on the surface of the crystal are much stronger than those inside the crystal. The surface metal bonds bear most of the load during a–b (elastic stage). Young’s modulus and yield stress of single crystal copper can be obtained based on the elastic stage, as shown in Fig. 2b. With the increase in temperature, both Young's modulus and yield stress show a linear decreasing trend, indicating that the resistance of single crystal copper to external deformation decreases with the increase in temperature. The b–c stage is the stress drop stage. The metal bond on the crystal surface first breaks, leading to necking, after the yield point (Point b) is reached in the tensile process. At 4 K temperature, the energy release of the surface metal bond after fracture is very significant, and the corresponding stress drop phenomenon also appears in the tensile curve. Stress drop is the embodiment of material brittleness; the greater the stress drop range, the more brittle the material. The c–d stage is the plastic strain stage in which the necking degree increases continuously until complete fracture. In this stage, the metal bonds on the crystal surface where necking occurs have been completely broken, and the load is borne by the metal bonds inside the crystal. Compared with the metal bond on the crystal surface, the strength of the metal bond inside the crystal is weak and its fracture process is relatively slow. This results in a fluctuating downward trend of stress in the tensile curve. The longer the c–d stage, the better the toughness of the material. It can be seen from Fig. 2a that even when the temperature is reduced to 4 K, single crystal copper has a certain toughness. The stress drop values of stress drop stage (b–c) and plastic strain of necking stage (c–d) at different temperatures are further extracted to study the variation of toughness and brittleness of single crystal copper at nanoscale with temperature, as shown in Fig. 2c. The stress drop amplitude of b–c stage decreases rapidly, when the temperature rises from 4 to 1200 K. The stress drop is 5.1 GPa at 4 K, but only 0.25 GPa at 1200 K, which is nearly 20 times different. On the other hand, when the temperature rises from 4 to 1200 K, the plastic strain increases gradually from 0.43 to 0.55. This shows that with the increase in temperature, the brittleness of single crystal copper material decreases and the toughness increases.

**Fig. 3:**
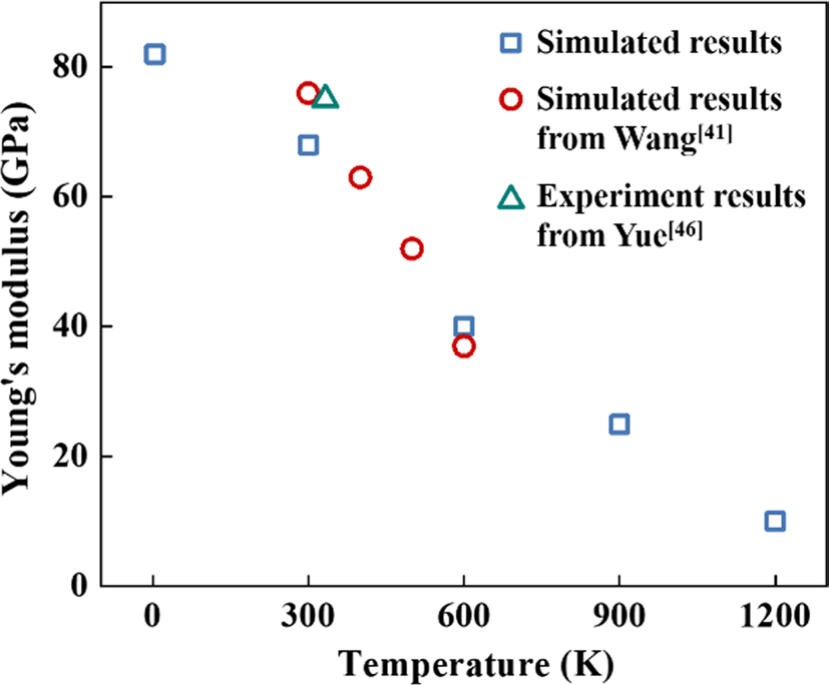
Comparison of Young’s modulus results

It should be pointed out that the stress–strain curves obtained in this work are basically consistent with those reported in the literatures under similar test conditions. The simulated Young’s modulus agrees well with the reported results [41, 44–46], as shown in Fig. 3. The excellent agreement validated the simulation approach and the MD model used in this study.

**Fig. 4:**
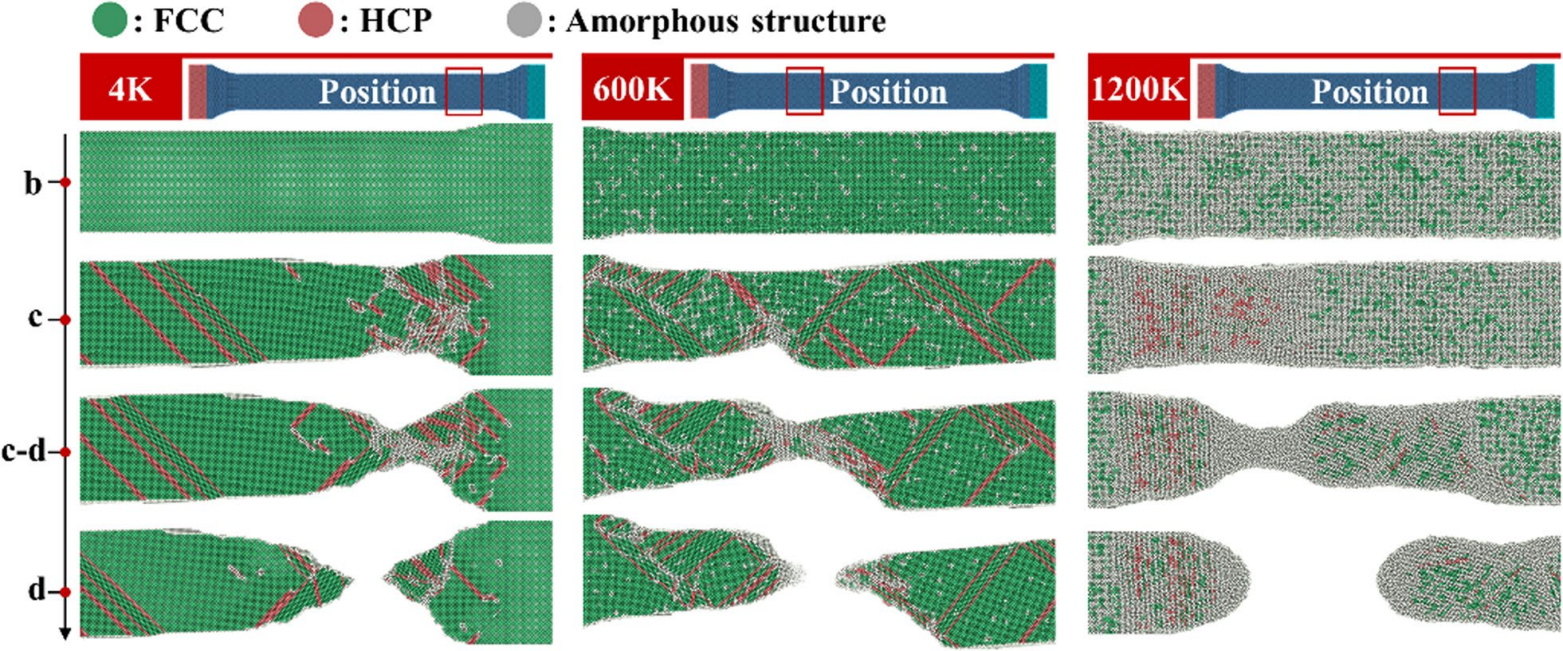
Crystal structure distribution at 4 K, 600 K and 1200 K

In order to obtain the crystal structure change characteristics of single crystal copper with different toughness and brittleness during tensile process, the common neighbor analysis method is used to study the crystal-type distribution during tensile process, as shown in Fig. 4. Atoms forming FCC (face-centered cube) structure are green, and FCC structure is also the initial crystal structure of single crystal copper crystal before stretching. The red part shows the HCP (hexagonal densest packing) crystal structure. The crystal structure of HCP is the crystal structure generated after the slip of adjacent FCC crystals caused by dislocation. The appearance of HCP crystal structure also reflects the toughness characteristics of materials. Gray atom is amorphous structure, its motion state is relatively free, and the ability to resist external deformation is poor, with strong plasticity and fluidity. At the temperature of 4 K, the crystal has a sound FCC structure at the maximum yield stress point b, and the metal bonds on the crystal surface do not break. When the tensile process reaches point c, the metal bond on the surface of the necking position is broken, and a small amount of HCP crystals are formed at the necking position. When the tensile process reaches c–d, the number of HCP crystals at the crystal necking increases and the amorphous structure begins to appear. At point d, the crystal breaks, and the fracture tip is sharp. When the temperature is 600 K, a small amount of amorphous structure appears inside the crystal at point b during the tensile process, which weakens the brittleness of the crystal. The sporadic distribution of amorphous structures resembles “defects” that induce the rupture of metallic bonds on the crystal surface. At point c, the metal bonds on the crystal surface have basically broken, and a large number of HCP crystals in the crystal are evenly distributed. Between c and d, the necking occurs at the position where the crystal density of HCP is the highest, and the amorphous structure mainly concentrates at the necking position. The crystal breaks at point d, and the sharpness of the fracture tip is lower than that at 4 K. At 1200 K, when the tensile process reaches point b, there are a large number of uniformly distributed amorphous structures in the crystal. At this time, the brittleness of crystal has basically disappeared and has strong toughness. When the tensile process reaches point C, the overall deformation of the crystal is still relatively uniform, and there is no obvious HCP crystal in the crystal. Between c and d, the necking occurs, and the necking is completely composed of amorphous atoms. At point d, the crustal breaks, and the tip of the fracture is a hemispherical shape similar to fluid.

In conclusion, at the nanoscale, the brittleness of single crystal copper is significant when it is reduced to 4 K, but it still has a certain toughness. With the increase in temperature, the brittleness of single crystal copper decreases and the toughness increases. When the temperature is raised to 1200 K, the brittleness of single crystal copper disappears, and it shows good plasticity and fluidity.

## The Mechanism of Nanometer Chip Formation

The chip formation mechanism of single crystal copper is studied, based on the toughness and brittleness characteristics of single crystal copper at different temperatures. Firstly, the MD model of nano-cutting at different temperatures is established. Then the crystal structure, shear strain, cutting force, atomic displacement vector and atomic movement track in the cutting process are analyzed. The chip formation mechanism under different toughness and brittleness is summarized.

### Cutting Model

**Fig. 5:**
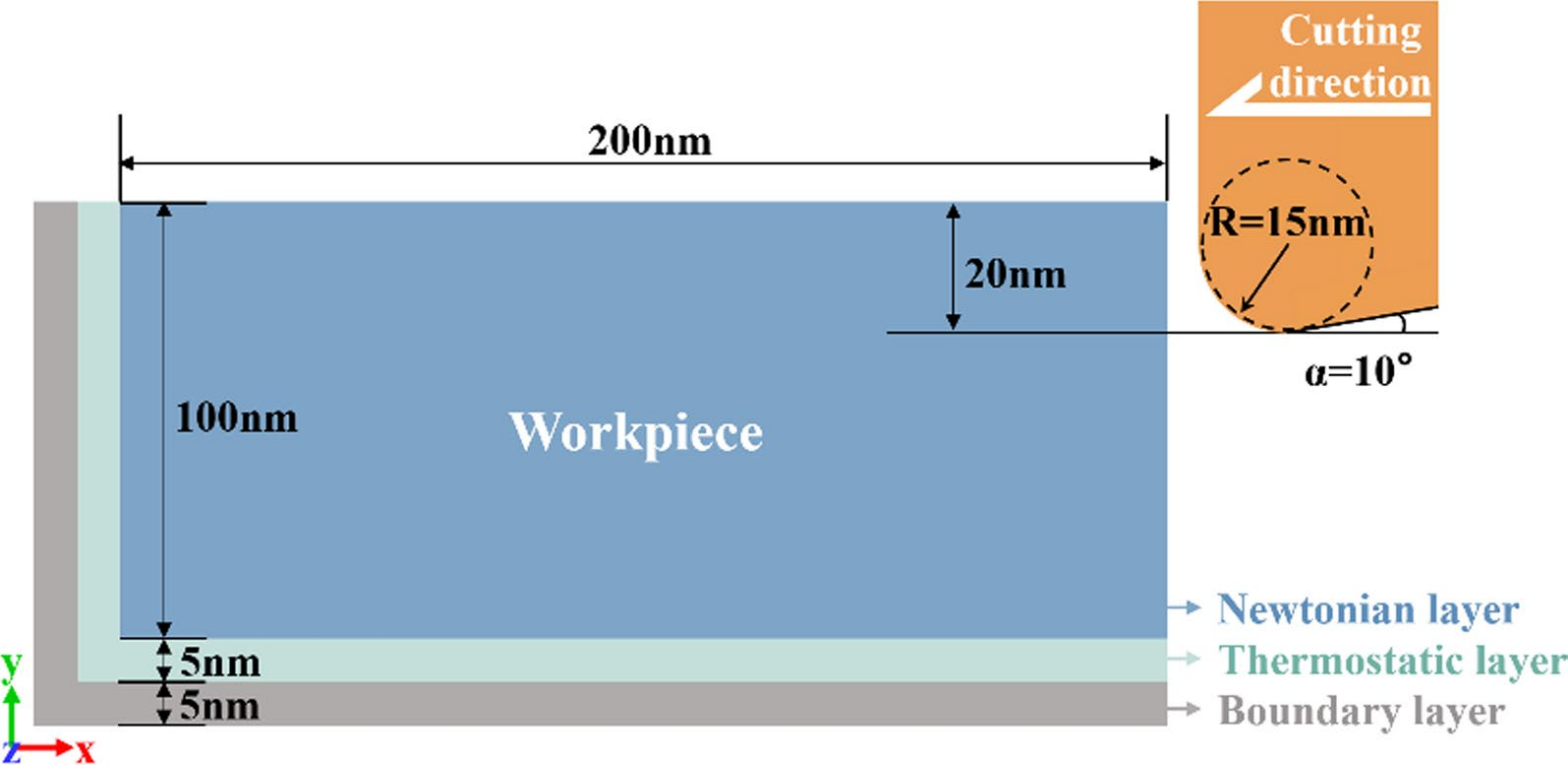
Nano-cutting model

MD model of nano-cutting is shown in Fig. 5. The workpieces of the model is composed of single crystal copper. The workpiece sizes in *x*, *y* and *z* directions are 210 nm, 110 nm and 1.06 nm, respectively. The boundary conditions of the model in *x* and *y* directions are fixed boundary, and in *z* direction is periodic boundary. The model is divided into Newtonian layer, Thermostatic layer and Boundary layer. Among them, the part to be cut is Newtonian layer. The Thermostatic layer plays a role in controlling the temperature of the workpiece. The workpiece is fixed by the Boundary layer. The tool is made of diamond (C) crystals. The tool rake angle is set as 0° to reduce the influence of the rake angle [47–50]. The clearance angle of the tool is 10°, and the negative tool back angle can effectively reduce the fluctuation of cutting force caused by friction [51]. In order to prevent the simulation results from being affected by the tool wear, the tool is set as a rigid body. The tool radius used in ultraprecision machining is usually between 10 and 100 nm [50, 52, 53]. In consideration of the computing capability and computing efficiency, a tool radius of 15 nm is selected, which is a little smaller than the cutting depth. The cutting depth and cutting speed are 20 nm and 100 m/s, respectively. The model contains three interaction potentials: Cu–Cu potential, Cu–C potential and C–C potential. The EAM potential is used for Cu–Cu interaction potential. Morse potential [54] is used for Cu–C interaction potential. Morse potential is widely used to describe the interaction between the diamond tool and metal workpiece in MD simulation of cutting. Tersoff potential [55], which can accurately describe the interaction between atoms in the covalent system, is selected for C–C interaction potential. Specific cutting parameters are shown in Table. 1. After the model is established, the conjugate gradient method and nose–Hoover method are used to relax the model successively. After relaxation, cutting simulation is performed. The NVE ensemble is used in cutting process. The time step of the simulation process is set to 1 fs. After the simulation is completed, Matlab and Ovito software is used for post-processing and visualization (Table 2).

**Table 2:** Specific parameters of nano-cutting model

Properties	Parameters
Interatomic interaction	EAM, Morse, Tersoff
Model size (*x*, *y*, *z*)	210 nm, 110 nm, 1.06 nm
Number of workpiece atoms	1,947,054
Tool rake/clearance angle	0°/10°
Tool edge radius	15 nm
Depth of cut	20 nm
Cutting speed	100 m/s
Cutting direction	$\left({001}\right)\left[\stackrel{\mathrm{-}}{1}{\text{00}}\right]$
Equilibration temperature	4 K, 300 K, 600 K, 900 K, 1200 K
Time step	1 fs

### Crystal Structure Characteristics of Atoms

**Fig. 6:**
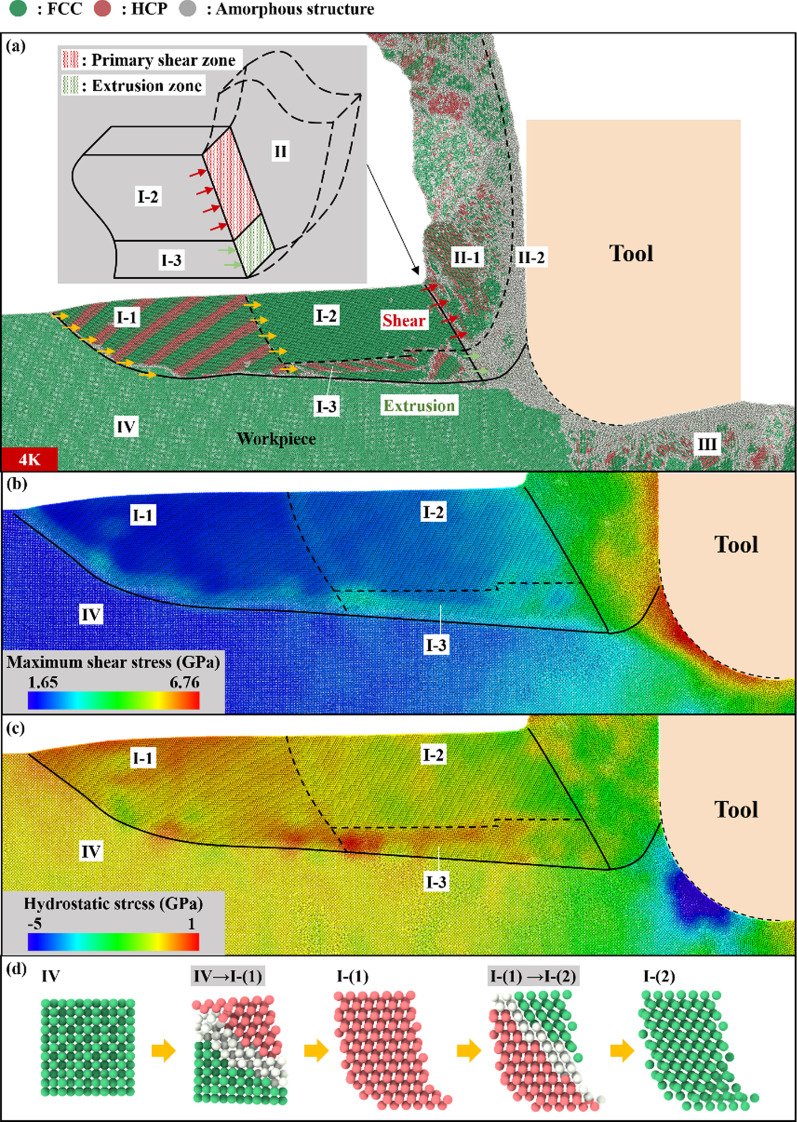
Morphology of chip root at 4 K, **a** crystal structure distribution, **b** crystal structure change process, **c** maximum shear stress distribution, **d** hydrostatic pressure distribution

**Fig. 7:**
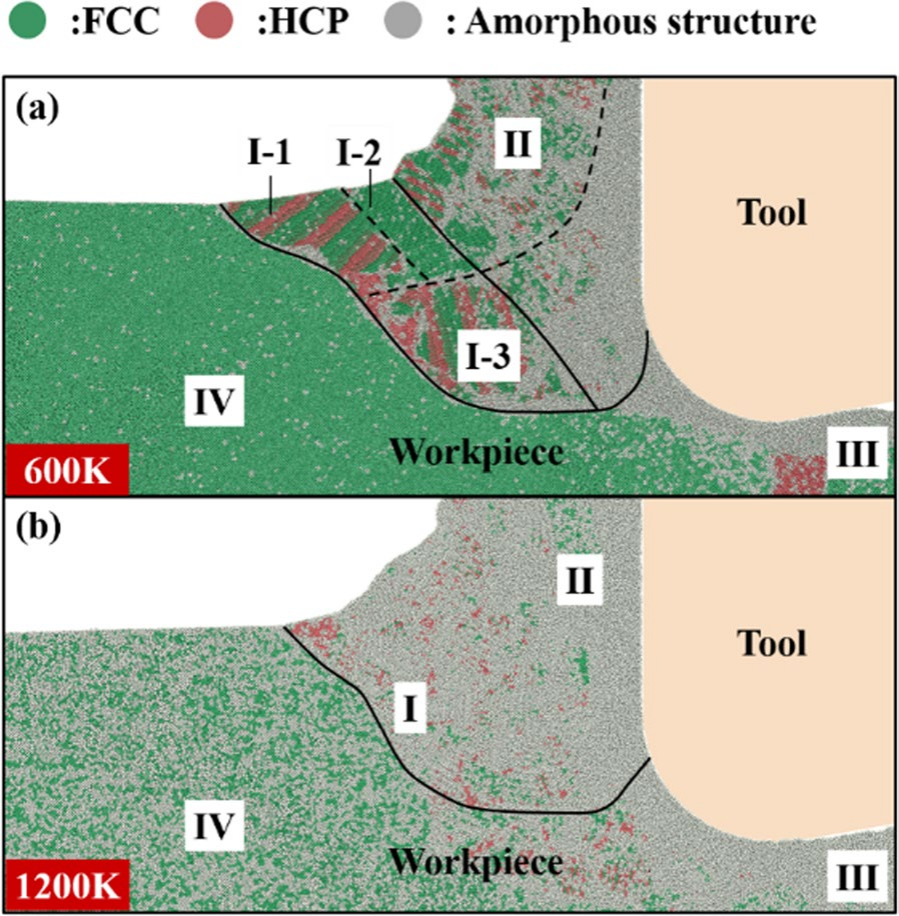
Crystal structure distribution of chip root, **a** 600 K, **b** 1200 K

**Fig. 8:**
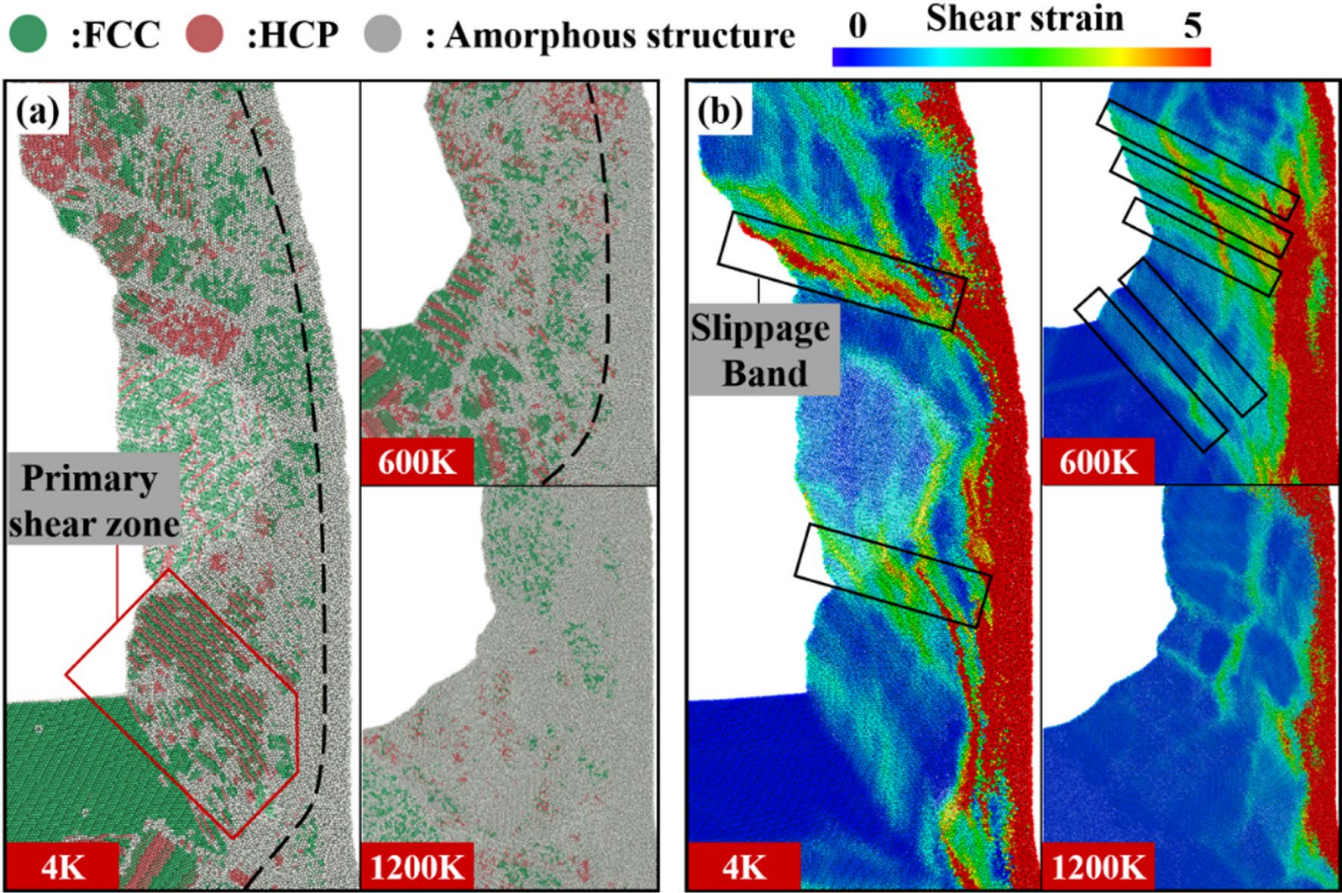
Chip morphology at different temperatures, **a** crystal structure, **b** shear strain nephogram

**Fig. 9:**
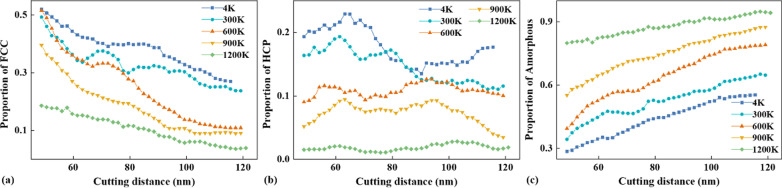
The proportions of atomic types plotted with cutting distance for different temperature, **a** FCC, **b** HCP, **c** amorphous

The chip formation process of single crystal copper with different toughness and brittleness is studied by Common Neighbor Analysis. First, the atoms in front of the tool that are affected by the tool load are analyzed. These atoms are about to form chips, and their crystal structure, stress and strain distribution are shown in Figs. 6 and 7. Then, for the atoms that have formed chips, the crystal structure distribution, shear strain nephogram and cutting force are analyzed, as shown in Figs. 8 and 9. A complete chip formation process is presented by describing various parameters before and after chip formation. It should be pointed out that Figs. 6, 7, 8 only give the crystal structures for the temperature of 4 K, 600 K, and 1200 K, because the crystal structure of 300 K is similar with that of 4 K, and the crystal structure of 900 K is similar with that of 1200 K. For simplicity, the crystal structure for the temperature of 300 K and 900 K is not shown in these figures.

When the cutting temperature is 4 K, according to the crystal structure distribution, the atoms in front of the tool can be divided into four zones: Tool influence zone (I), Chip (II), Machined surface (III) and To-be-machined zone (IV), after the cutting process is stable, as shown in Fig. 6a. The Tool influence zone (I) can be further subdivided into three regions: the region of parallel distribution of banded HCP structures (I-1), FCC crystal region (I-2) and the region of mixed distribution of multiple crystal structures (I-3). At 4 K, the brittleness of the material is obvious, and the influence range of the cutting tool is large. The shear stress at the junction of the Tool influence zone (I) and the To-be-machined zone (IV) is high, as shown in Fig. 6b. The Tool influence zone (I) tends to slip along the I–IV boundary and detach from the workpiece as a whole. However, it should be pointed out that the material in the front end of the zone (I-1) and the To-be-machined zone (IV) extrude each other, under the action of the tool. The distribution of hydrostatic stress near the I–IV boundary is not uniform, as shown in Fig. 6c, which leads to shear slip along the (${1}$ ${1}$ $\overline{{1}}$) plane. This results in the formation of some parallel banded HCP structures at the zone (I-1). The shear slip during the formation of HCP releases some of the energy. Therefore, the shear stress near the I–IV boundary cannot reach the threshold that separates the Tool influence zone (I) completely from the workpiece. Meanwhile, the hydrostatic stress distribution in the middle of the zone (I-2) is relatively uniform. The zone (I-2) is uniformly compressed along the direction of the tool propulsion, and the crystals structure in the zone (I-2) remains FCC structure. As the cutting tool advances in the cutting process, atoms in the To-be-machined zone (IV) continue to flow into the zone (I-1). Some of the crystals in zone (IV) slip and change into the HCP structure. This part of the crystal will undergo further dislocation before it flows into the (I-2) zone, and the crystal structure will change from HCP to FCC again, as shown in Fig. 6d. The atoms below the zone (I-1) flow into the zone (I-3). These atoms exhibit a hybrid distribution of HCP, FCC and amorphous structure under the combined action of shear and extrusion. Chip (II) can be divided into two areas. The area near the tool-chip interface presents an amorphous structure. The area near the chip-free surface presents a block crystal structure separated by amorphous bands. During chip formation, FCC crystals in the zone (I-2) undergo periodic shear slip along the primary shear zone. Then the chips with alternating FCC and HCP crystal structures are formed. The atoms in the zone (I-3), which are about to form chips, are transformed into amorphous structure under the extrusion of the tool, the block chip and other atoms in the zone (I-3). Then the amorphous atoms move up along the front edge of the tool to form flowing chips. The Machined surface (III) can be roughly divided into two areas. The surface atoms are amorphous structures with a certain thickness. The subsurface atoms present a state of mixed distribution of various crystal structures. The To-be-machined zone (IV) is less affected by the tool and retains the original FCC crystal structure.

Crystal structure distribution at 600 K and 1200 K is shown in Fig. 7. At 600 K, a small number of amorphous atoms are scattered in the initial crystal. The brittleness of the material decreases and the toughness increases compared with that at 4 K. This significantly reduces the width of the Tool influence zone (I). The zone (I) also no longer has a tendency to detach from the workpiece. The reduction in FCC crystal size in (I-2) zone leads to the reduction in the size of the block chip formed by shear. On the other hand, the reduction in the Tool influence zone (I) makes it more difficult to offset the uneven distribution of stress at the I–IV boundary. This also means that the force state and the crystal structure of the zone (I) becomes more complex. As a result, the thickness of the zone (I-3) increases, and the number of flowing chips generated by extrusion increases correspondingly. At 1200 K, a large number of amorphous atoms have been distributed in the initial crystal. The brittleness of the material disappears. The material shows good fluidity. At this time, the Tool influence zone (I) and To-be-machined zone (IV) can still be separated by amorphous atoms. But the Tool influence zone (I) and the Chip (II) are indistinguishable. Block chips basically disappear, and most of the chips are formed by extrusion. The chip formation process is approximately in a flowing state.

The core of the chip formation process is how the atoms in the zone (I-2) and zone (I-3) are separated from the workpiece under the shear and extrusion. During the chip formation by shearing, the continuous advance of the tool brings the FCC crystal in the zone (I-2) closer to the primary shear zone. Adjacent FCC crystal layers near the main shear zone undergo small shear slip constantly, resulting in relatively uniform shear plastic deformation. Shear slip results in the conversion of FCC crystals into HCP crystals, releasing some of the energy generated by tool propulsion. After the formation of HCP crystal, the crystal strength increases. So that the shear slip cannot continue in the HCP crystal. Then, new shear slips occur in other FCC crystals near the primary shear zone, releasing energy continuously. However, the input energy of the tool cannot be completely release by the shear slip, and the energy will accumulate continuously. The crystal will have a long-distance shear slippage in the primary shear zone, releasing the remaining energy and generating a slippage band, when the energy is accumulated to a certain extent. Atoms in slippage band become amorphous by friction. The above processes are periodic and eventually form sawtooth chips similar to those at the macroscale [56]. In the process of chip formation by extrusion, the atoms entering the extrusion zone are transformed into amorphous structure under the combined action of the tool, the block chip and the stagnation region. As the extrusion process continues, amorphous atoms move upward to form chips.

Figure 8 shows chip crystal structure distribution and the corresponding shear strain nephogram at different temperatures. At lower temperatures, more energy is required for shear slip on the primary shear zone, and each shear slip has a better offset effect on the tool propulsion energy. Therefore, the lower the temperature is, the less the residual energy after each crystal shear slip is, and the slower the energy accumulation rate is, when the tool pushes the same distance and transfers the same energy to the workpiece. In addition, the energy threshold required for slippage at lower temperatures is higher. Therefore, it takes longer time to accumulate energy for large slippage at low temperature. Slippage bands also form at a lower frequency. At 4 K, the temperature is low and a large number of shear slips are generated through the primary shear zone. Parallel HCP structures caused by shear slips are shown in Fig. 8a. The distance between slippage bands is large. The spacing of amorphous bands caused by slippage is also large. At this temperature, amorphous chips are distributed on the right side of block chips and their thickness is narrow. When the temperature is 600 K, the brittleness of the material decreases. During chip formation, the energy accumulation rate increases, and the energy threshold for dislocation decreases, resulting in a decrease in the spacing of slippage bands. As dislocation occurs more frequently, the degree of slippage decreases, and the shear strain value at the slippage band decreases compared with that at 4 K. The spacing of large shear deformation caused by slippage also decreases, as shown in Fig. 8b. At this temperature, the thickness of amorphous chips on the front surface of the tool increases, accounting for 1/4 of the total chip thickness. When the temperature rises to 1200 K, the material exhibits strong toughness. The energy threshold required for slippage is significantly reduced. Each shear slip is basically accompanied by slippage. But the degree of slippage is very small, the degree of deformation caused by shear slip and slippage is almost the same. So, the shear strain during chip formation is uniform and there is no obvious slippage band in the chip. At this temperature, the thickness of amorphous chips increases significantly and the block chips disappear. The amorphous chips formed by extrusion account for more than 80% of the total chips. The proportions of the FCC, HCP and amorphous atoms in the uncut chip evolving with cutting distance are shown in Fig. 9. It can be seen that, as the tool moves ahead, the proportion of the FCC atoms decreases, while the proportion of the amorphous atoms increases. Besides, with the increase in temperature, the proportions of both FCC and HCP atoms decrease, but the proportion of amorphous atoms increases. The total proportion of FCC and HCP atoms is much larger than that of the amorphous atoms at low temperature, but the proportion of the amorphous atoms becomes much larger at high temperature.

**Fig. 10:**
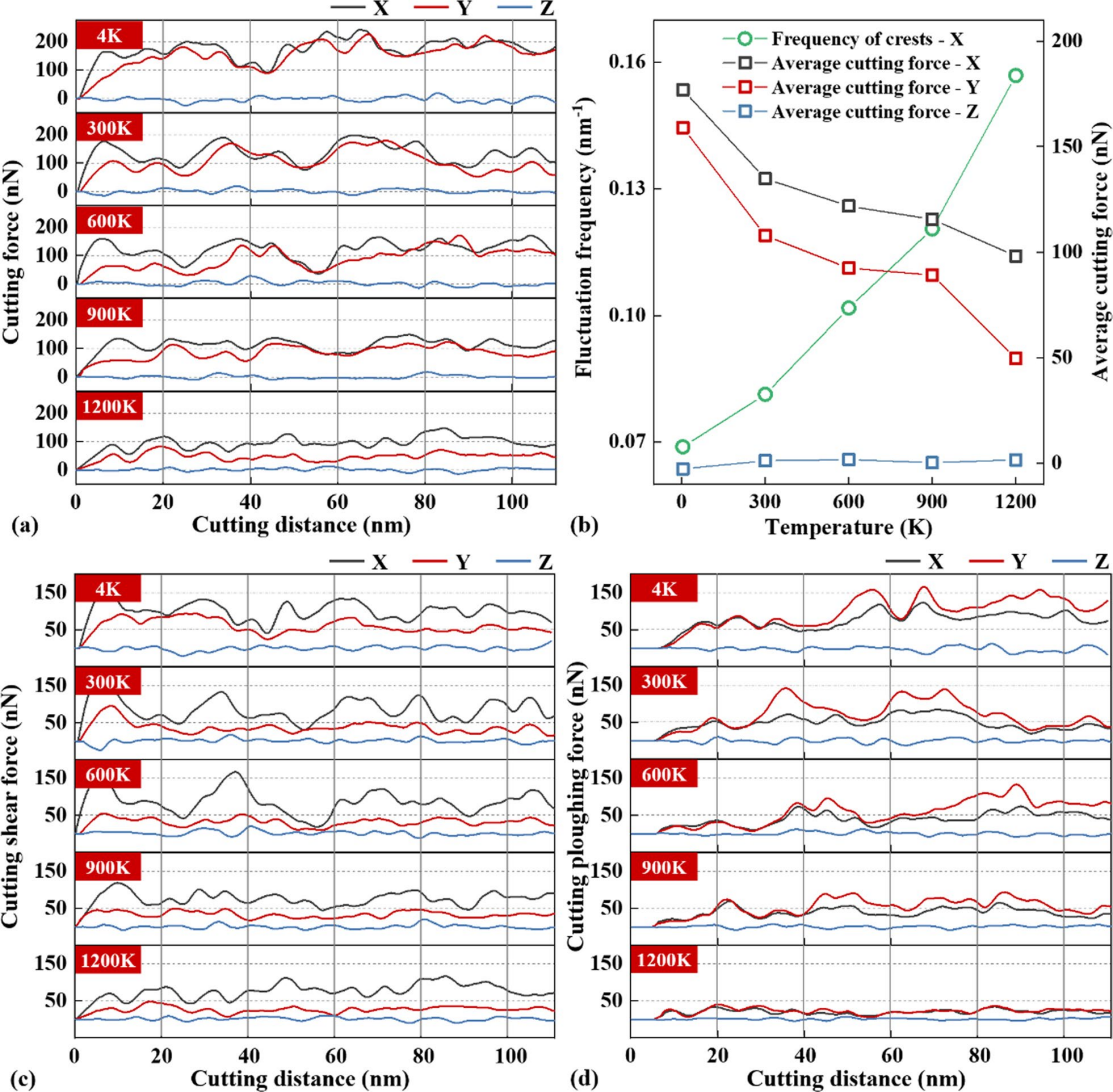
Characteristics of cutting forces at different temperatures, **a** cutting force, **b** average cutting force and fluctuation frequency, **c** cutting shear force, **d** cutting plowing force

Shear slip and slippage during chip formation can also be reflected in the fluctuation of cutting force. However, many factors, such as tool friction and atomic collision, will lead to a small disturbance of cutting force. In order to make the change of cutting force more intuitive, the curve is smoothed. After eliminating the disturbance, the evolutions of cutting force(CF), average cutting force(ACF) and the frequency of cutting force fluctuation(FCF) at different temperatures are shown in Fig. 10a, b. It can be seen from Fig. 10a that the CFs in *X* and *Y* directions decrease gradually with the increase in cutting temperature, but the FCF in *X* direction increases with the increase in temperature. The CF in the *Z* direction fluctuates around zero and is almost independent on the temperature. For the CF curve, each small fluctuation of the CF represents a shear slip. Each shear slip offsets some, but not all, of the energy generated by the tool propulsion. As for the temperature of 4 K, the strength of the material is high and the toughness is weak. The ACF is 184 nN, and the difference between the peak and valley of the CF is about 140 nN. This means the energy released by each slippage is large, and the threshold energy required for slippage is relative high. For this condition, the fluctuation frequency of FCF is low, indicating a large slippage space. When the temperature increases to 600 K, the toughness of the material increases. The value of ACF decreases to 144 nN, and the difference between peak and valley of the CF decreases to 90 nN. It indicates that the threshold energy required for the emergence of slippage is reduced. On the other hand, the FCF increases, which means the energy accumulation rate increases and the slippage spacing decreases. When the temperature is up to 1200 K, the brittleness of the material disappears and the material shows strong toughness. The value of ACF reduces to 98 nN. The amplitude of the fluctuation of FCF becomes very small, but the fluctuation frequency increases greatly. It implies that the amount of energy required for slippage becomes very small. The slippage occurs uniformly in the process of chip formation.

**Fig. 11:**
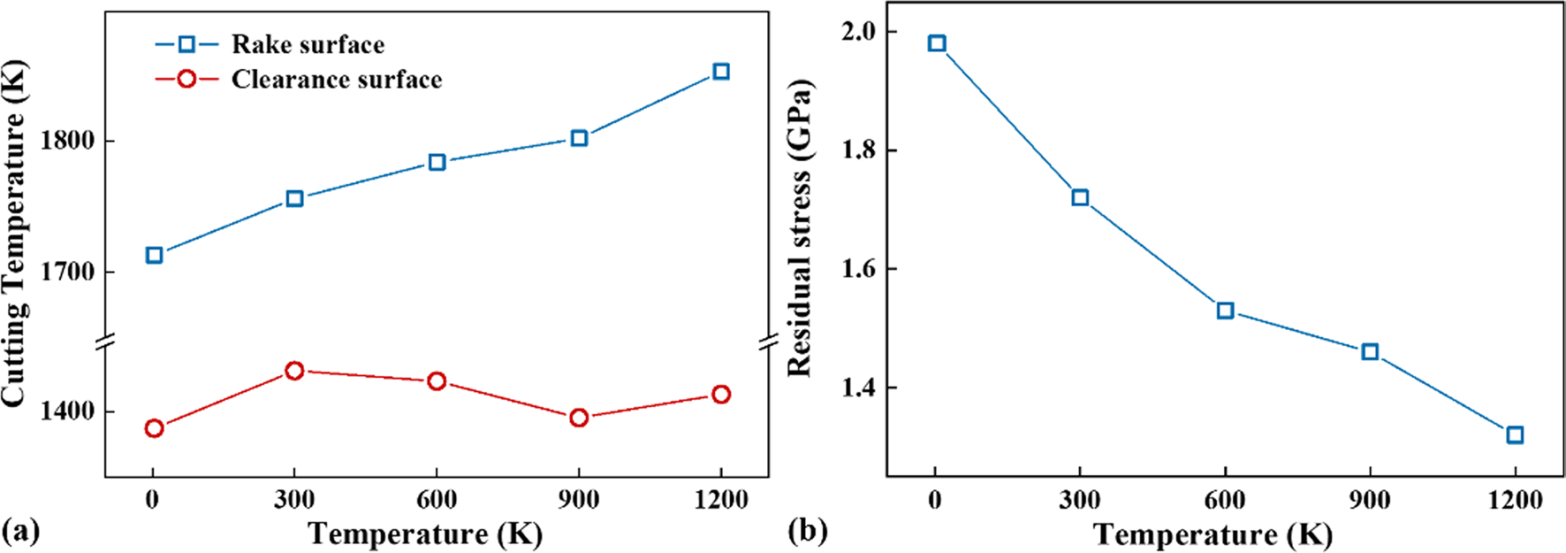
Maximum tool temperature and residual stress at different temperatures, **a** tool temperature on rake surface and clearance surface, **b** residual stress on the machined surface

The ratio of the cutting depth to the tool tip radius is close to one, there will be a stagnation zone near the tool tip [57]. The cutting shear force (CSF) located onto the cutting edge above the stagnation point and the cutting plowing force (CPF) located onto the cutting edge below the stagnation point are further obtained, which are given in Fig. 10c, d, respectively. It can be seen that both the CSF and CPF decrease with the cutting temperature, and the fluctuation frequency of the CSF and CPF also increase with the temperature. In addition, the maximum tool temperature on the rake surface and clearance surface, as well as the residual stress on the machined surface, are further investigated, as shown in Fig. 11. It can be seen from Fig. 11a that, with the increase in cutting temperature, the maximum temperature on the rake surface rises from 1700 to 1870 K, but the maximum temperature on the clearance surface is almost unchanged. As for the residual stress on the machined surface, it decreases with increasing the cutting temperature, see Fig. 11b.

### Motion Characteristics of Atoms

**Fig. 12:**
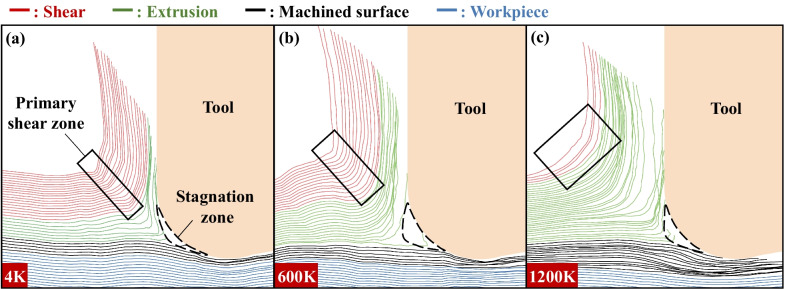
Atomic motion trajectory **a** 4 K, **b** 600 K, **c** 1200 K

**Fig. 13:**
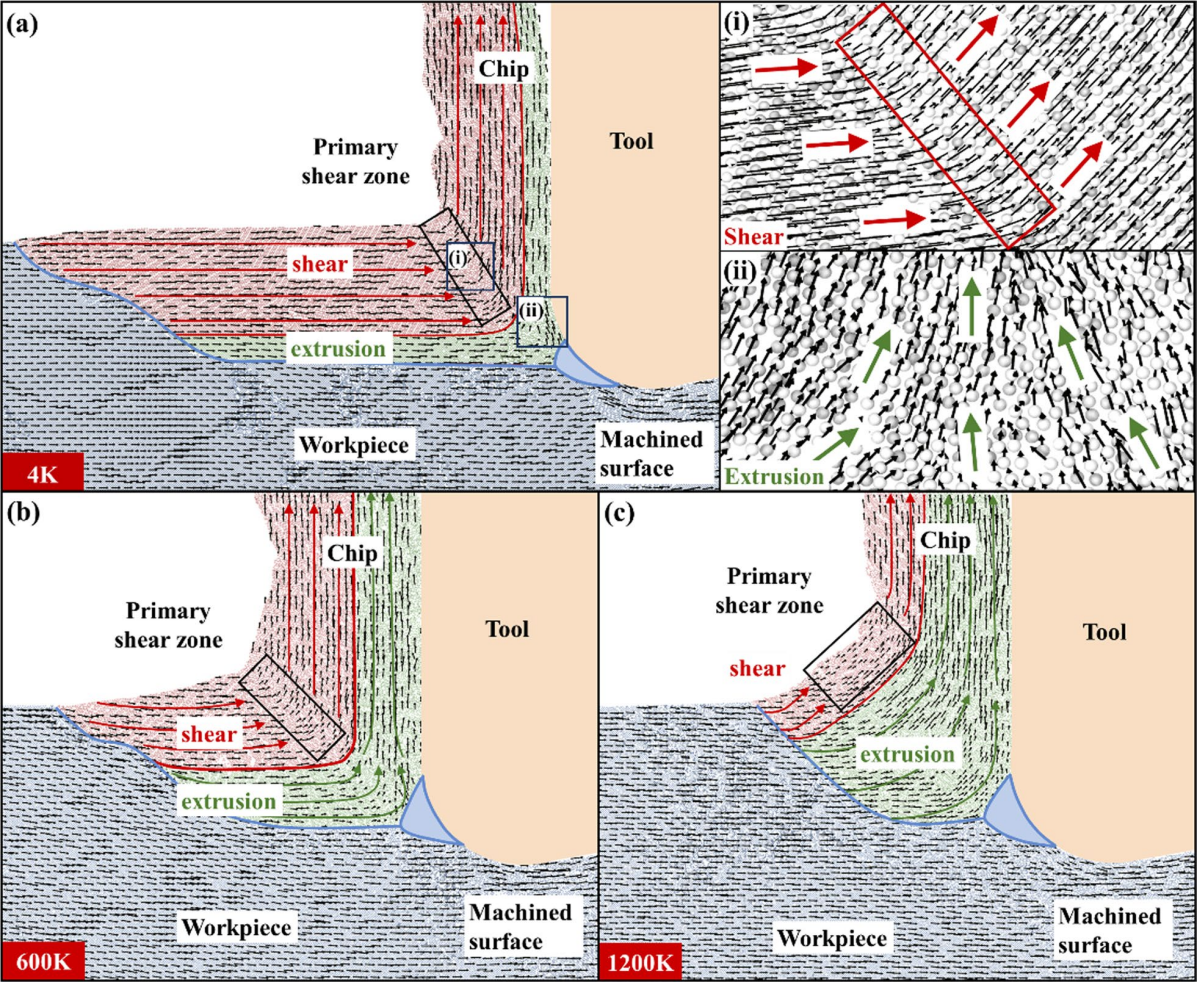
Atomic displacement vector diagram **a** 4 K, **b** 600 K, **c** 1200 K

It is found that chip formation can be divided into shear and extrusion, based on the crystal structure analysis. But there is still a lack of evidence for the classification of chips formed by different ways. Thus, the atomic motion trajectory and atomic displacement vector during chip formation are analyzed respectively, as shown in Figs. 12 and 13. The differences of chip formation are further analyzed.

First, take a row of atoms and calculate their atomic movement relative to the tool. It is found that chip, machined surfaces and stagnation region can be clearly distinguished by atomic motion trajectory at different temperatures, as shown in Fig. 12 There is almost no trajectories in the triangular stagnation region in front of the tool. The chip trajectory and the machined surface trajectory are divided into two directions after passing through the stagnation region. The chip trajectory can be further divided into shear and extrusion parts. The shear trajectory remains relatively parallel until chip formation. Then, the shear trajectory is turned in the primary shear zone under the action of the tool. At this time, parallel state of shear trajectory is destroyed and the spacing changes. But there is no overlapping or interleaving phenomenon of shear trajectory. After the chip is formed, the shear trajectory returns to a relatively parallel state again. The extrusion trajectory also remains relatively parallel until chip formation. However, when the extrusion trajectory turns in the primary shear zone, the trajectory spacing decreases obviously. Overlapping and interleaving phenomena also occur. After chip formation, the extrusion trajectory cannot change into a relatively parallel state. The trajectories of the atoms forming the machined surface are only overlapped under the tool. The trajectories of workpiece atoms are basically not affected by the tool and always remain relatively parallel. When the temperature is 4 K, the number of shear trajectories is larger than that of extrusion trajectories. The turning radius of the chip trajectory is small when its direction changes. The width of the primary shear zone is narrow. At 600 K, the number of shear trajectories decreases. Overlap and interlacing are more obvious in the extrusion trajectory. The turning radius of the chip trajectory in front of the tool increases. The width of primary shear zone also increases. When the temperature is 1200 K, the proportion of extrusion trajectory in chip trajectory increases greatly. The turning radius of chip trajectory increases further and becomes an arc shape. The width of the primary shear zone increases significantly.

Shear and extrusion can be further distinguished by atomic displacement vectors. Taking the tool as a reference frame, the atomic displacement vectors at different temperatures are shown in Fig. 13 Based on the characteristics of atomic displacement vector, the vector in the figure can be divided into five parts: chip formed by shearing, chip formed by extrusion, workpiece, stagnation region and machined surface. The atomic displacement vector changes when crossing the boundary between the processing zone (IV) and the tool affected zone (I) mentioned above. Therefore, the boundary between I and IV area is used as the boundary between chip vector and workpiece vector. The distribution of atomic displacement vector is similar to that of atomic movement trajectory. The stagnation region divides the displacement vector into chip vector and machined surface vector. Chip vector can be further divided into shear vector and extrusion vector. The variation of shear vector is parallel → turn → parallel. The variation of extrusion vector is parallel → extrusion → parallel. When the temperature is 4 K, there are more atoms forming chips by shearing, and the shear vector direction changes in the primary shear zone, as shown in Fig. 13(a-i). The number of atoms forming chips by extrusion is small. The displacement vectors of these atoms are convergent due to the extrusion of the tool and stagnation region, as shown in Fig. 13(a-ii). At 600 K, the toughness of the material increases. The width of the primary shear zone increases. But the number of shear vectors is reduced. At 1200 K, the brittleness of the material disappears and the material shows strong toughness. The proportion of chips formed by shearing decreases, the proportion of chips formed by extrusion increases, and the width of the primary shear zone increases correspondingly.

### Chip Formation Mechanism

**Fig. 14:**
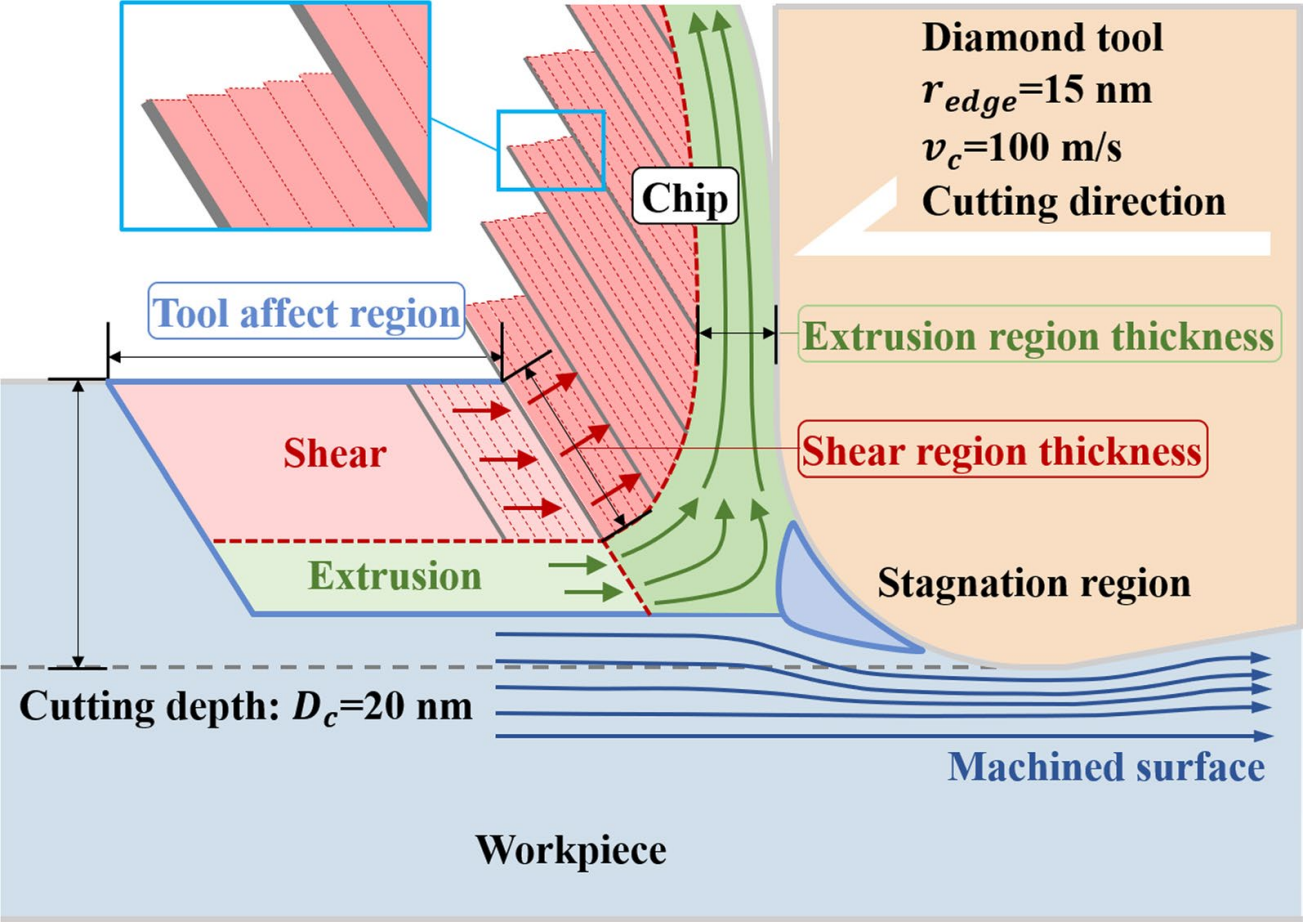
Schematic diagram of chip formation by shearing and extrusion

In summary, the chip formation process is shown in Fig. 14. The atoms of the workpiece within a certain distance from the tool are affected by the tool load, so their motion characteristics change. There is a dark blue triangular Stagnation zone at the tip of the tool, where the atoms are stationary relative to the tool and travel with the tool. During the cutting process, the tip of the stagnation zone divides the workpiece atomic flow into two parts: the machined surface and the chip. Atoms flowing down through the tip of the stagnation zone are rubbed and squeezed at the bottom of the tool to form the machined surface. The atoms moving upward through the tip of the stagnation zone will be separated from the workpiece to form chips. The atoms near the free surface form block chips under shearing (red area), and the workpiece atoms near the knife-chip interface form flowing chips under extrusion (green area). Small shear slip occurs between adjacent crystals during block chip formation, as shown by the red dotted line. When the shear slip reaches a certain amount, large slippage will occur between adjacent block chips, as shown in the figure. The cutting direction of the chip is always from the chip root to the free surface, so the small shear slip direction in the block chip and the long-distance slippage between the block chip are parallel to the primary shear plane. With the increase in temperature, the frequency of long-distance slippage increases, but the amplitude of slippage decreases. The flowing chips are amorphous, and they are FCC, HCP and amorphous before forming. During the cutting process, these atoms are pushed and rubbed by the cutting tool, stagnation zone and the upper block chip, and the crystal structure is destroyed to form amorphous structure. The direction of its movement also changes, and it flows upward along the tool in a convergent manner, eventually forming the flowing chip. With the increase in the initial temperature, the toughness of the material increases, the yield stress decreases, the frequency of the slippage (gray solid line) increases, and the size of the block chip decreases. Rising temperatures also make it easier for materials to transition from crystalline to amorphous structures. Therefore, the thickness of block chip formed by shearing decreases and the thickness of amorphous flow chip formed by extrusion increases.

## Conclusion

In this paper, nano-tensile simulation and nano-cutting simulation of single crystal copper at different temperatures are carried out, and chip formation mechanism of single crystal copper at different toughness and brittleness is studied in depth. The main conclusions are as follows:

1. The results of nano-tensile simulation show that the single crystal copper still has a certain toughness at the nanoscale when the temperature is reduced to 4 K. The results of nano-tensile simulation show that the single crystal copper still has a certain toughness at the nanoscale when the temperature is reduced to 4 K. With the increase in initial temperature, the stress drop degree of tensile stress–strain curve decreases and the plastic strain increases after reaching the yield point, which indicates that the toughness of the workpiece material increases and the brittleness decreases.
2. In nano-cutting simulation, chip formation can be divided into two types: shear and extrusion. The workpiece material near the free surface has shear slip and periodic long-distance slippage along the primary shear direction, forming block chips. With the increase in temperature, the formation frequency of the long-distance slippage increases, but the slippage amplitude decreases. The crystal structure of the workpiece material near the tool-chip interface is destroyed by the extrusion of the tool, the stagnation zone and the block chip above, forming amorphous flowing chip.
3. The results of particle trajectory and particle displacement vector analysis show that with the increase in initial temperature, the thickness of block chip formed by shearing decreases, while the thickness of flowing chip formed by extrusion increases.
4. With the increase in cutting temperature, the proportions of both FCC and HCP atoms decrease, but the proportion of amorphous atoms increases, the average cutting force decreases, the fluctuation of the cutting force becomes weaker, but the fluctuation frequency increases. Besides, both the shear force and plowing force decrease with the cutting temperature. Moreover, with increasing the cutting temperature, the maximum temperature on the rake surface rises, the maximum temperature on the clearance surface remains almost unchanged, and the residual stress on the machined surface decreases.

It should be pointed out that, due to the limitation of computing capability, the cutting depth adopted in this work is much smaller than that applied in the actual nano-cutting experiments. So the experimental verification of the MD model is unfeasible. We will increase the scale of the MD model of nano-cutting and validate it by the experimental results in our future work. Besides, the chip formation mechanism depends on the depth of cut. The mechanism of chip formation under different cutting depths, especially under the depth lower than the cutting edge radius, will be carefully investigated in the future, since the depth of cut is very often lower than the cutting edge radius during ultraprecision cutting.

## Data Availability

The data that support the findings of this study are available from the corresponding author upon request.
